# Phenology modulates the top-down control of ants on bird ectoparasites: from mutualism to antagonism

**DOI:** 10.1038/s42003-025-09387-9

**Published:** 2025-12-22

**Authors:** Jesús M. Avilés, Ángela Salido, Joaquín L. Reyes-López, Deseada Parejo

**Affiliations:** 1 Departamento de Ecología Funcional y Evolutiva, EEZA-CSIC, Almería, Spain; 2 Unidad Asociada CSIC-UNEX Ecología del Antropoceno, Badajoz, Spain; 3 Departamento de Botánica, Ecología y Fisiología Vegetal, Universidad de Córdoba, Córdoba, España

**Keywords:** Behavioural ecology, Zoology

## Abstract

Bird–ant interactions are diverse but rarely tested experimentally, limiting their integration into ecological theory. One hypothesized but unverified benefit is ant-mediated parasite control in bird nests. Here, we present the first experimental evidence supporting this hypothesis in a wild system involving house sparrows (*Passer domesticus*), arboreal ants (*Crematogaster scutellaris*), and blood-feeding mites (*Pellonyssus reedi*). Using field ant-exclusion experiments, we show that ant presence reduces mite abundance and enhances chick growth early in the breeding season, but has detrimental effects later. Nestlings in ant-excluded nests also show consistently higher H/L ratios, indicating greater physiological stress. Structural equation modeling reveals that ant effects on nestling condition are indirect and mediated by mite load. Our findings provide the first causal demonstration of ant-mediated parasite suppression in birds, revealing that the outcome of this interaction is highly context-dependent. This work underscores the dynamic nature of species interactions and highlights overlooked ecological roles of ants in avian systems.

## Abstract (web summary)

Ant presence in house sparrow nests reduces blood-feeding mites, boosts early chick growth, and lowers stress, but effects vary seasonally, revealing context-dependent ant-mediated parasite control.

## Introduction

Birds and ants are among the most ubiquitous taxa in terrestrial ecosystems worldwide. While a variety of interactions between them have been documented, our understanding of their ecological and functional significance remains largely anecdotal and poorly integrated^1^. Existing literature documents a broad spectrum of trophic and ecological relationships, ranging from reciprocal predation—where certain bird species prey on ants^2,3^, and ants, in turn, prey on bird eggs or nestlings^4,5^—to behaviors such as active and passive anting, in which birds either rub ants to their plumage or allow ants to move freely over their bodies^6,7^. Competitive interactions have also been reported, involving shared trophic resources such as invertebrate prey^8,9^,seeds^10,11^, or fruit^12^, as well as nesting sites—including tree cavities and open nests—where ants may forage on nest-dwelling invertebrates or organic residues such as feathers and droppings^13,14^. Ants may also benefit from the warm microclimatic conditions of bird nests^15^, which can facilitate the development of ant larvae under thermally favorable environments^16^. In Tropical regions, numerous bird species associate with swarm-raiding army ants, exploiting arthropods flushed out by the ant activity^17–19^. Additionally, ants may confer protective benefits against predation to birds, as suggested by increased daily survival rates of avian nests located in ant-inhabited trees^20^. Despite this diversity of known interactions, many potential ecological roles of ants in avian life histories remain underexplored. One such underappreciated dynamic involves the possible role of ants in mediating ectoparasite loads within bird nests.

Building upon this foundation, emerging evidence suggests that ants may play a role in regulating ectoparasite loads within bird nests—an interaction that could confer indirect benefits to both organisms and lead to forms of mutualism. For example, observational evidence has shown that several ant species occupy the cavity nests of the European roller *Coracias garrulus* during the breeding season and prey on both adult and larval stages of the ectoparasitic fly *Carnus hemapterus*^21^. Similarly, ants belonging to the genera *Myrmica* and *Lasius* have been observed preying on *Protocalliphora azurea*, a blowfly ectoparasite of wood warbler *Phylloscopus sibilatrix* nests^22^. In addition, a study by Brown et al.^23^ found that *Crematogaster lineolata* and *Formica* spp. ants reduced the number of visible swallow bugs by 74–90% on active nests of cliff swallows, *Petrochelidon pyrrhonota*. However, this reduction had no detectable effect on the reproductive success of cliff swallows, suggesting a commensal interaction^23^. In such instances, birds may benefit from ant-mediated parasite suppression, while ants may exploit avian-derived food resources or prey on the parasites. Although this mutualistic scenario is plausible, these studies did not confirm that the presence of ants controlled the parasitic load on chicks, thus emphasizing the need for experimental validation.

A major challenge in fully understanding bird–ant–ectoparasite interactions lies in their strong context dependence^24,25^. Ecological factors such as temperature, nutrient availability, predator and parasite densities^26–28^, as well as the relative abundances of the interacting species^29,30^ can all influence the nature and outcome of these interactions. Moreover, individual traits of the birds, such as age, size, or condition, may further modulate these dynamics by affecting their likelihood of interacting with ants or their susceptibility to ectoparasites^31,32^. Previous studies have shown that competitive interactions between pied flycatchers *Ficedula hypoleuca* and wood ants *Formica aquilonia* can shift toward facilitation when predation risk is high^33^. However, in the specific case of ant-mediated parasite control, this interaction has yet to be examined experimentally—particularly with respect to its potential variation across ecological contexts. This gap highlights the need for further research into how environmental and biological factors shape the outcomes of bird–ant–ectoparasite interactions, which may range along a continuum from antagonism to mutualism^32^.

In this study, we investigate the context-dependent interactions among house sparrows *Passer domesticus*, the arboreal ant *Crematogaster scutellaris*, and the blood-feeding mite *Pellonyssus reedi*, an ectoparasite known to impair nestling development^34,35^. Ant–house sparrow associations were common in the study area during the breeding season, with ants detected in 36.1% of nests in 2024 and in 47.36% of 152 nests in 2025. Using an experimental field design, we test whether excluding *C. scutellaris* from sparrow nests affects mite infestation and, consequently, nestling growth, body condition, and physiological stress. We consider phenology a key factor that could modulate the outcome of the interaction between sparrows and ants, as both ectoparasite loads and parental condition are likely to vary throughout the breeding season^36,37^, potentially influencing these interactions. Indeed, previous studies on House sparrows and *Pellonyssus* mites have shown a marked seasonal effect on infestation, with nestlings from third breeding attempts carrying significantly higher mite burdens than those from second broods, likely because the mite’s short (5–7 day) life cycle enables several generations to build up within nests and persist across successive breeding attempts^38^. Our central hypothesis posits that the presence of ants reduces mite burden on nestlings, thereby improving their somatic growth and lowering physiological stress, with stronger effects expected in early-season broods, when ant-mediated mite suppression is likely to be most effective due to the lower mite infestation^39^. To evaluate this, we measured ectoparasite load, nestling growth metrics, and the heterophil-to-lymphocyte (H/L) ratio as a stress indicator. Specifically, we predict: (1) lower mite abundance in ant-inhabited nests; (2) enhanced nestling growth and lower physiological stress in the presence of ants; (3) increased mite loads in late broods, especially in ant-exclusion nests; (4) stronger ant-related benefits in early-season broods, since high parasite loads may exceed the ants’ capacity for control, making their positive effects more evident when parasite pressure is lower and more manageable.

These predictions are based on the assumption that mite pressure increases seasonally and that ants provide consistent antiparasitic control by feeding on mites. Although direct evidence is lacking for this species, *Crematogaster* ants are omnivores known to consume a broad range of tiny invertebrates^40^, and experiments have shown that ants can regulate mite populations through predation^41,42^. In addition, *Lasius* ants have been documented feeding on *Dermanyssus* mites—close relatives of *Pellonyssus*—that primarily infest chickens and other birds^43^, suggesting that incidental predation on similar taxa by *C. scutellaris* may also occur. By integrating experimental manipulation, seasonal variation, and physiological measures, this study aims to elucidate how bird–ant–ectoparasite interactions shift along the mutualism-antagonism continuum depending on the ecological context. Finally, to disentangle the direct and indirect effects of ant exclusion on mite load and nestling mass, we employ structural equation modeling (SEM), which allows simultaneous evaluation of multiple causal pathways.

This study provides the first experimental evidence that arboreal ants can suppress parasite loads in wild bird nests. Ant presence lowered the abundance of blood-feeding mites and improved early-season chick growth, although ant effects became detrimental later in the season. Nestlings in ant-excluded nests exhibited higher H/L ratios, suggesting increased physiological stress. SEM showed that ant influences on nestling condition were indirect and operated through changes in mite load. Overall, the study demonstrates that ant-mediated parasite control is real but strongly context-dependent, highlighting the dynamic nature of bird–ant interactions and revealing an underappreciated ecological role of ants in avian breeding systems.

## Results and discussion

### Effect of removal on ant presence

Ants were present in only 3 of 40 ant-exclusion nests (7.5%) compared to 13 of 36 control nests (36.1%) at chick banding. This difference was significant (Fisher’s exact test, *p* = 0.004), suggesting that the exclusion treatment was effective in reducing ant presence. Occasional observations of ants in some exclusion nests, and their absence in a few controls, suggest the treatment’s effectiveness was not absolute, likely making comparisons conservative since any true differences between treatments would be underestimated. Importantly, the exclusion treatment did not substantially affect other ground-dwelling invertebrates aside from sporadic occurrences of earwigs (*Forficula* spp.), confirming *Crematogaster* ants as the primary taxa influenced.

Although ant presence in control nests at fledging was confirmed in fewer than half of the cases, our estimates are likely conservative due to brief nest visits, the prioritization of nestling monitoring, and observations often conducted when ants are inactive (activity peaks around midday, whereas many nests were checked from 8 a.m.). The alternative explanation that the glue used in experimental boxes stressed parents and thereby increased mite infestation seems unlikely, as all boxes were handled identically, the glue was colorless and odorless, and neither the nest nor the box would have been directly affected. While minor undetected factors—such as the presence of earwigs or other scavenger species not observed during nest visits—cannot be entirely ruled out, ant presence remains the most plausible factor driving treatment differences.

### Ant removal effects on reproductive parameters

Ant exclusion did not significantly influence the number of chicks that hatched or the number of chicks that successfully fledged. The mean number of hatchlings was similar between treatments (control: 1.52 ± 0.09; ant-exclusion: 1.53 ± 0.09; Poisson GLMM with hatchling number as the response, treatment as a fixed factor, and laying date as a covariate: *F*₁,₄₉ = 0.11, *p* = 0.74), and the interaction between treatment and laying date was not significant (*F*₁,₄₉ = 0.10, *p* = 0.75; Supplementary Table 1). Likewise, fledging success showed no evidence of a treatment effect (control: 1.48 ± 0.10; ant-exclusion: 1.54 ± 0.09; Poisson GLMM with number of fledglings as the response, treatment as a fixed factor, and laying date as a covariate: *F*₁,₄₈ < 0.01, *p* = 0.96), and again the treatment × laying date interaction was not significant (*F*₁,₄₈ = 0.01, *p* = 0.90; Supplementary Table 1).

### Ant removal effects on mites and nestling development

**Fig. 1:**
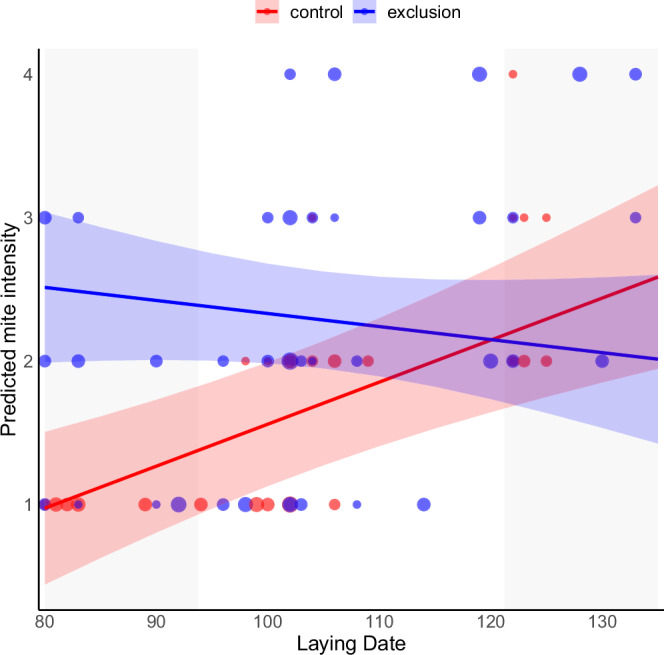
Effect of ant removal on mite intensity across the breeding season. Predicted mite intensity of House sparrows in relation to experimental ant removal. Shaded ribbons represent 95% confidence intervals for different experimental groups. The predicted values and confidence intervals are derived from the ordinal mixed-effects regression model reported in Supplementary Table 1. Gray background bands highlight early (below the first quartile) and late (above the third quartile) laying periods. Mite intensity was scored using a four-point ordinal scale: 1 = no mites, 2 = 1–10 mites, 3 = 20–30 mites, and 4 = > 30 mites. Data points show raw individual observations, with size scaled according to the number of observations for each combination of laying date and mite intensity; larger points indicate more frequently observed values. *N* = 196 nestlings from 43 nests.

The exclusion of ants significantly impacted mite intensity depending on house sparrow phenology (GLMM: *F*₁,₄₃.₇ = 6.36, *p* = 0.015, Supplementary Table 2). In early nests, removing ants caused a marked increase in mite loads on chicks, supporting our first prediction that mite abundance would be lower in ant-inhabited nests. However, as the season progressed, mite load tended to converge between treatments, with exclusion values falling within the control’s confidence intervals by about day 110 (Fig. 1), indicating that ants reduce mite intensity during roughly the first half of the breeding season. Control nests showed a significant seasonal increase in mite intensity, reaching the baseline levels observed in exclusion nests (estimate = 0.033, SE = 0.005, *F*₁,₆₄ = 45.56, *p* < 0.0001). This trend was absent when ants were excluded (estimate = –0.013, SE = 0.009, *F*₁,₁₀₉.₃ = 2.09, *p* = 0.151), indicating that ant presence disrupts the natural seasonal rise in mite infestation. These findings align with previous suggestions of ant-mediated parasite control in nests^22,23^, and extend them through experimental evidence.

**Fig. 2:**
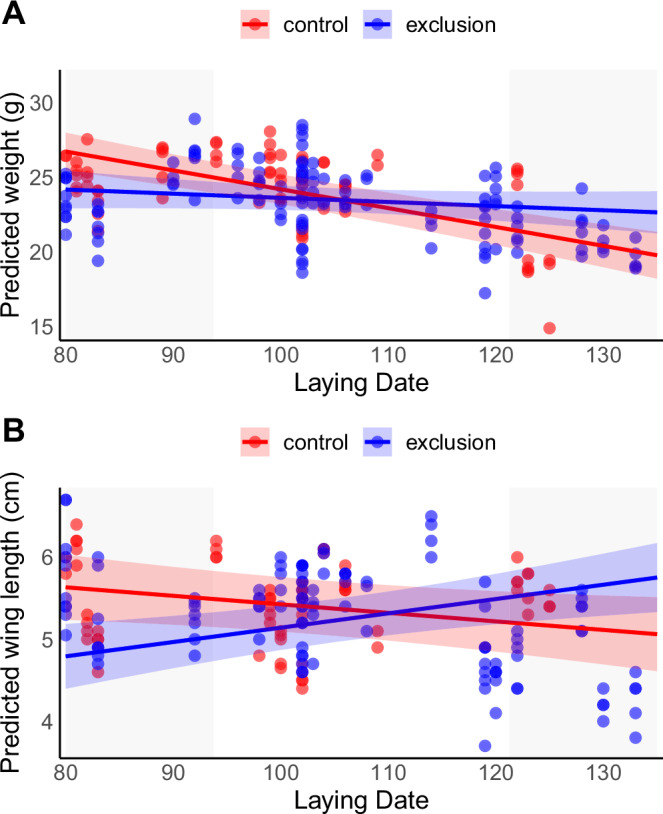
Effect of ant removal on chick growth across the breeding season. Predicted chick weight (**A**) and wing length (**B**) across the breeding season of House sparrows in relation to experimental ant removal. Shaded ribbons represent 95% confidence intervals for different experimental groups. The predicted values and confidence intervals are derived from the linear mixed-effects models reported in Supplementary Table 2 and Supplementary Table 3, respectively. Gray background bands highlight early (below the first quartile) and late (above the third quartile) laying periods. Data points show raw individual observations. *N* = 196 nestlings from 43 nests for chick weight and *N* = 183 nestlings from 43 nests for wing length.

Chick weight in House Sparrows tended to decrease as the season progressed (GLMM: estimate = –0.028, SE = 0.02, *F*_1,107.7_ = 28.43, *p* < 0.0001; Supplementary Table 3, Fig. 2A). However, this seasonal decline was significantly influenced by ant exclusion (GLMM: *F*_1,169.1_ = 14.58, *p* = 0.0002; Supplementary Table 3). Specifically, the reduction in weight was much more pronounced in control nests (GLMM: estimate = –0.136, SE = 0.017, *F*_1,67.03_ = 67.09, *p* < 0.0001) compared to ant-exclusion nests (GLMM: estimate = –0.041, SE = 0.019, *F*_1,76.37_ = 4.57, *p* = 0.036). As a result, chicks from early nests weighed less when ants were excluded, whereas in late nests, the pattern was reversed (Fig. 2A).

A similar interactive effect of treatment and phenology was observed for chick wing size (GLMM: *F* = 15.44, *p* < 0.0001; Supplementary Table 4). In control nests, wing length decreased significantly with laying date (GLMM: *β* = -0.012, *F*_1,54.91_ = 24.67, *p* < 0.001), whereas in ant-excluded nests, wing length showed a non-significant increasing trend over the season (GLMM: *β* = 0.011, *F*_1,66.64_ = 3.16, *p* = 0.08). Thus, the presence of ants promoted longer wings in early broods, but appeared to constrain wing growth in later ones (Supplementary Table 4, Fig. 2B), echoing the context-dependent nature of this interaction.

Interestingly, the inflection points for chick weight and wing length appears to coincide with that of mite intensity (around day 110 of the breeding season) (Figs. 1 and 2). This may indicate a threshold mite load (approximately intensity score 2) beyond which ants no longer confer a protective effect on house sparrows. Additionally, nest ID was significant in all three models, suggesting strong nest-level differences in mite load, chick weight, and wing length. This implies that factors specific to each nest—such as microhabitat conditions, parental quality, or initial parasite exposure—can strongly influence chick development and parasite infestation, highlighting the importance of accounting for nest-level variability in our analyses.

**Fig. 3:**
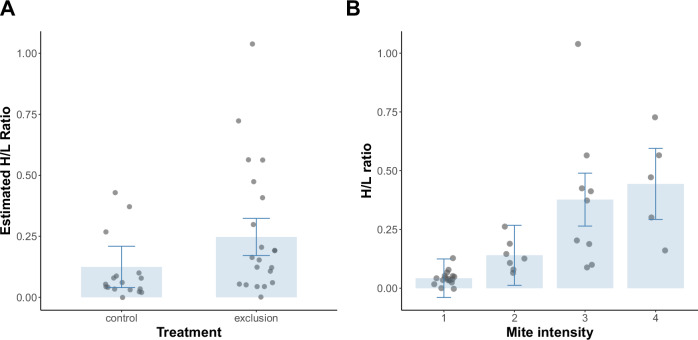
Effects of ant exclusion and mite intensity on chick H/L ratios. **A** Estimated effect of ant exclusion treatment on H/L ratio. Bars represent model-derived means for each treatment group, with error bars indicating 95% confidence intervals. “Ant exclusion” refers to nests where ants were experimentally excluded, and “control” refers to unmanipulated nests. The H/L ratio was significantly lower in control chicks compared to those from ant exclusion nests (*p* = 0.036). Estimates are based on a linear model controlling for laying date and chick body weight. **B** Relationship between mite intensity and H/L ratio. Bars represent estimated means per mite intensity category with 95% confidence intervals. Data points show raw individual observations. *N* = 38 nestlings from 38 nests.

Physiologically, ant exclusion increased the heterophil/lymphocyte (H/L) ratio of chicks, indicating elevated stress (estimate = –0.123, *p* = 0.036; Supplementary Table 5, Fig. 3A). Laying date was also positively correlated with H/L ratio (estimate = 0.0074, *p* = 0.002; Supplementary Table 5), consistent with higher stress in late-hatched chicks regardless of treatment. Moreover, H/L ratios rose with mite intensity (estimate = 0.14, *F*_1,36_ = 36.18, *p* < 0.001; Fig. 3B), suggesting that the reduction of physiological stress in control nests may stem from ant-mediated suppression of mites, as hypothesized under our second prediction.

### Structural equation modeling of ant exclusion effects

**Fig. 4:**
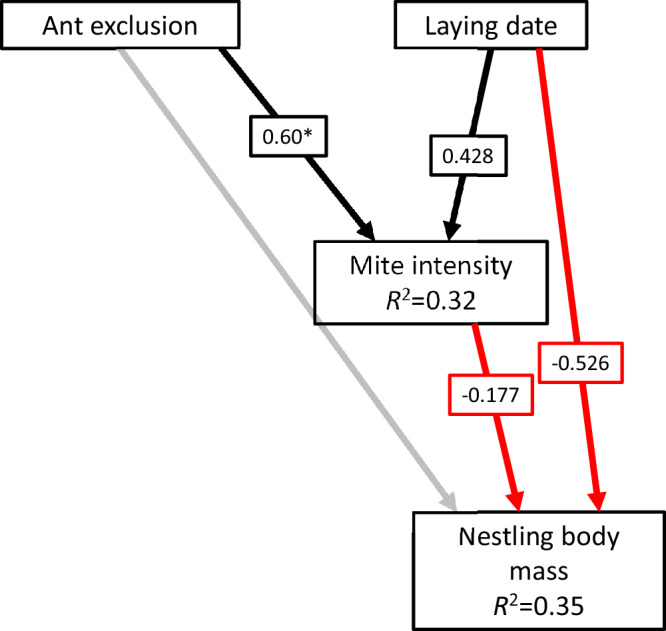
Structural equation model of ant removal, mite load, and chick body mass. Arrows represent hypothesized unidirectional relationships among variables. Black arrows indicate significant positive effects (*p* < 0.05), and red arrows indicate significant negative effects. Paths with non-significant effects (*p* ≥ 0.05) are shown as semi-transparent arrows. *R*² values (marginal) from the component models are displayed inside the boxes of the response variables. All coefficients are standardized, except for the effect of ant exclusion treatment, which represents the difference between the exclusion and control groups and is marked with an asterisk (*). The model was fitted using piecewise SEM, including nest ID as a random effect. *N* = 196 nestlings from 43 nests.

SEM revealed that mite intensity increased with later laying dates (standardized estimate = 0.43, *p* < 0.001) and was significantly higher in ant-exclusion nests (estimate = 0.60, *p* < 0.01; Fig. 4). Mite intensity negatively affected chick weight (estimate = –0.18, *p* = 0.03), and laying date also reduced weight (estimate = –0.53, *p* < 0.001). The direct effect of ant exclusion on chick weight was non-significant (*p* = 0.82), suggesting that ant benefits were mediated primarily through mite reduction rather than direct effects on nestlings. The model explained 32% of variance in mite intensity and 35% in chick weight; including nest ID as a random effect improved model fit substantially (conditional *R*² = 0.80 for mite intensity, 0.79 for chick weight), highlighting strong among-nest differences. This SEM outcome aligns with our fourth prediction that ant-related benefits are amplified early in the season when parasite burdens are lower. Our results indicate that ant exclusion led to a rapid increase in mite loads early in the season, whereas control nests showed a gradual seasonal rise. The apparent lack of further increase in mite intensity in exclusion nests may reflect a “ceiling effect”: in the absence of ants, mite populations reach high densities early, leaving little scope for additional seasonal growth. In contrast, in nests with ants, mite loads are initially suppressed, allowing a gradual increase over the season. However, our GLMMs showed that as the season progressed, even high mite burdens in late broods could not be suppressed by ants, which coincided with poorer developmental outcomes—supporting our third prediction that mite loads peak in late-season broods. This interpretation reconciles the GLMM and SEM results, highlighting that ant-mediated effects are strongest early in the season when parasite burdens are low, and that the seasonal pattern of mite intensity depends on the ants’ ability to control nascent populations.

### Mechanisms and context-dependence of ant–bird interactions

These results are consistent with the hypothesis that *Crematogaster* ants provide an antiparasitic service by reducing ectoparasite burdens, with positive cascading effects on avian growth and stress physiology. Other potential mechanisms include disruption of parasite life cycles or microenvironmental changes within the nest. Future research using behavioral observations, experimental feeding trials, or gut content analyses could clarify the underlying mechanisms. Despite initial mutualistic benefits, the relationship shifted across the breeding season. In late nests, mite loads were high regardless of treatment, and ant presence no longer improved—sometimes even worsened—nestling growth metrics. This shift supports our interpretation of a mutualism–antagonism continuum, echoing Haemig’s^33^ findings of context-dependent outcomes in ant–bird interactions. Our study uniquely demonstrates this continuum within a single host species across a temporal gradient, revealing that ant-related costs—possibly from disturbance, aggression, or competition—may increase late in the season. We have recently observed occasional remains of *Crematogaster* ant mandibles on injured toes and commissures of sparrow chicks, which may indicate disturbance. This observation opens the possibility of investigating in future studies how such potential disturbances vary with ectoparasite presence and across the breeding season. Seasonal increases in temperature may also contribute by modulating ant behavior, activity, and dominance within the nest environment. Temperature variation is a key factor shaping ant community structure in Mediterranean environments^44,45^. Indeed, community-level studies have shown that dominance status—and thus the degree of interspecific ant interactions—shifts with temperature fluctuations^46^. Moreover, late-breeding sparrows are often of lower quality (e.g., younger or subordinate individuals), potentially reducing their ability to mitigate the negative impacts of both ectoparasites and ants^47^. The feeding habits of *C. scutellaris* may also vary seasonally in response to changing nutritional needs at both the individual and colony level^48,49^. In spring, during periods of low activity and early larval development, colonies tend to prioritize protein intake, while in summer, at peak activity, carbohydrates and water become the main dietary focus^50^. Colony phenology thus shapes workers’ foraging behavior. Thus, the interaction between ants and sparrows cannot be fully understood without considering this broader ecological context.

Taken together, our findings provide compelling evidence that ant–bird interactions are not fixed, but dynamically shaped by ecological context. Crematogaster ants can act as mutualistic ectoparasite controllers early in the season, but their net effect on avian fitness becomes neutral or antagonistic later—likely due to ecological constraints on parasite suppression and/or increased disturbance or competition. While ant exclusion did not affect reproductive output (number of eggs, hatchlings, or fledglings), it did influence chick development, suggesting that ants may exert sublethal or quality effects on offspring rather than affecting their quantity or survival. This context-dependence underscores the importance of considering environmental gradients and multiple fitness components when evaluating the outcomes of interspecific interactions. By combining experimental manipulation, physiological assessment, and temporal variation within a single host species, this study advances our understanding of the conditional nature of mutualism and highlights the value of tractable systems like the *Crematogaster*–house sparrow association for studying the ecological and evolutionary mechanisms driving variability in species interactions.

## Materials and methods

### Study area

The study was conducted at the Valdesequera Experimental Station, a 200-ha open holm oak *Quercus ilex* woodland located near Badajoz, Extremadura, Spain (39°03′N, 6°48′W), managed by CICYTEX (Scientific and Technological Research Center of Extremadura). Approximately 300 cork nest-boxes with 3.2 cm entrance holes and predator-deterrent tubes are installed on the property. Each spring, these boxes are used for breeding by around 40 pairs of tits (great tits *Parus major* and blue tits *Cyanistes caeruleus*) and more than 100 pairs of house sparrows. At the beginning of each study season in February, all nest-boxes are cleaned to remove residual nesting material from the previous breeding season.

The region has a Mediterranean climate, with cold, rainy winters and hot, extremely dry summers.

### Life cycle of the mite

*P. reedi* is an obligate hematophagous mite of the suborder Mesostigmata, characterized by a hemimetabolous, wingless life cycle that develops entirely within bird nests^51^. It has been documented in the nests of various passerines, including the house sparrow^38,39,51^. The species undergoes four developmental stages, all completed in the nesting material. Non-feeding forms (larvae and deuteronymphs) remain within the nest substrate, whereas the blood-feeding stages (protonymphs and adults) alternate between residing in the nest and emerging periodically to feed on nestlings^51^; adults may also parasitize fledged birds^51^. The life cycle is brief, and reproduction is closely synchronized with chick growth, allowing populations to reach high densities under favorable conditions^38^.

### Ant presence monitoring in nest-boxes

In 2024, to quantify ant presence in nest cavities, all nest-boxes were inspected at least twice between March 23 and April 18. During each visit, we recorded the presence of *C.* *scutellaris* workers patrolling either the interior or the outer walls of the nest-boxes, as well as the presence of a nest of this species. Nests were identified by the steady traffic of workers through a crevice in the cork structure, a characteristic scorpion-like defensive posture in response to nest-box manipulation, or the direct transport of larvae by workers.

### Experimental setup

In the same year, an experiment was conducted to assess the effects of ant exclusion on mite abundance and chick development in house sparrows breeding in the nest-boxes. Nest-boxes were visited at a minimum frequency of once per week starting in the third week of March. During these weekly checks, as boxes containing sparrow eggs were detected, they were randomly assigned to one of two treatments: (1) Ant exclusion: the metal wire suspending the nest-box from holm oak (*Quercus ilex*) branches was coated with an insect glue (RASQUIM, Altres, Sevilla, Spain). This glue is odorless, colorless, tasteless, and non-toxic, and has been shown to be effective in preventing access by ground-dwelling invertebrates, such as ants, to suspended baits^52^. (2) Control: the metal-wire remained untreated, allowing ants unrestricted access to the nest-box. The glue was reapplied every 4 days to maintain its effectiveness, and the absence of ants in nest-boxes was verified at each visit. Nest failure was unaffected by treatment: 28% of control (10/36) and 30% of experimental nests (12/40) failed (*χ*²₁ = 0.05, *p* = 0.831). At the start of the experiment, ant activity was very low due to low ambient temperature; consequently, ants were only detected in the boxes that contained an ant nest, and hence treatment allocation was effectively blind to the presence of ants. Importantly, as previously described for parids^13^ and pied flycatchers^33^, nest-boxes with active ant nests were avoided by house sparrows and therefore were not included in the experiment (none of the 10 boxes that harbored an ant nest prior to the onset of sparrow breeding were selected). Thus, we can rule out the possibility that our results were confounded by sparrows of differing quality selectively avoiding ant-infested nest-boxes.

A total of 76 randomly selected nests that progressed to breeding were included in the study (40 in the ant-exclusion group and 36 in the control group). There were no significant differences in the mean treatment application dates between groups (control: April 19 ± 2.96 days [mean ± SE]; ant-exclusion: April 21 ± 2.81 days; GLM with treatment date as the dependent variable and treatment as a fixed effect: *F*₁,₇₄ = 0.18, *p* = 0.67). Likewise, laying dates (control: April 10 ± 2.46 days; ant-exclusion: April 11 ± 2.34 days; GLM with laying date as the dependent variable and treatment as a fixed effect: *F*₁,₇₄ = 0.15, *p* = 0.69) and clutch sizes (control: 5.47 ± 0.16 eggs; ant-exclusion: 5.38 ± 0.15 eggs; Poisson GLM with clutch size as the dependent variable and treatment as a fixed effect: *F*₁,₇₃ = 0.03, *p* = 0.87) did not differ significantly between groups. These results indicate that the two treatment groups were comparable in terms of reproductive timing and early parental investment, supporting the effectiveness of the randomization procedure.

On average, the ant-exclusion treatment was applied 10 days after the onset of egg-laying. In our population, *P. domesticus* lays between 3 and 8 eggs, with clutch sizes of 5–6 being most common (Supplementary Data). Females lay one egg per day, and incubation begins with the penultimate or last egg, lasting 11–12 days^53^; thus, by the time chicks hatch, the ant-exclusion treatment had been active for approximately five days on average. Given that chicks fledge around 14 days post-hatching^53^, and that *P. reedi* mites have a life cycle of 5–7 days^54^, two to three mite generations could occur within a single nestling period of the house sparrow host.

On day 12 post-hatching, chicks from both experimental groups were banded with metal rings, and a series of morphometric and physiological measurements were taken. Body mass was recorded using a Pesola spring balance (±0.5 g), tarsus length was measured with a digital caliper (±0.01 mm), and wing length was measured with a ruler (±0.1 cm). At the same time, mite load was visually estimated for each chick by examining body regions where ectoparasites typically aggregate, including the base of the feathers, cloacal area, and the inner surfaces of the wings and neck. Mite intensity was scored using a four-point ordinal scale: 1 = no mites, 2 = 1–10 mites, 3 = 20–30 mites, and 4 = > 30 mites. This method provides a rapid and reliable assessment of mite burden when direct counts are impractical due to the small size and high mobility of the parasites^55^.

Additionally, to assess physiological stress, the heterophil-to-lymphocyte (H/L) ratio was measured in a randomly selected subsample of 38 nests. Blood sampling was conducted blind with respect to the ant-exclusion treatment, comprising 17 control nests and 21 exclusion nests distributed across the entire breeding season. On the same day as banding, a blood sample was collected from one chick per nest as a representative sample for brood-level stress. The chick was randomly selected from the nest due to the barely noticeable differences in size within the broods of this species. Blood was drawn from the brachial vein, after banding and measuring, and smears were prepared, fixed, and stained with Wright-Giemsa stain. The H/L ratio was calculated by counting 100 leukocytes under oil immersion (1000×) and dividing the number of heterophils by the number of lymphocytes. This ratio is a widely used indicator of stress in birds, with higher values associated with increased glucocorticoid levels and greater mortality risk^56–58^.

The study was approved by the CSIC Committee for the Evaluation of Projects and Procedures Involving Animals for Experimentation. We complied with all relevant ethical regulations, with permits from the Junta de Extremadura for nest access.

### Statistical methods

We analyzed the effects of experimental ant exclusion on reproductive success at the nest level and on chick traits using models implemented in SAS (v9.4, SAS Institute Inc., Cary, NC, USA). Given that nestling conditions markedly deteriorate due to the decline in food resources in our study area^59^, as well as documented seasonal variation in mite loads^39^ and parental quality^36,37^, laying date was treated as a key ecological gradient. All models included laying date as a covariate, as well as its interaction with treatment, to test whether the effects of ant exclusion varied across the breeding season.

For reproductive success at the nest level, we fitted two generalized linear models (GLMs) with a Poisson error distribution and a log link function using the PROC GLIMMIX procedure. The first model examined the number of chicks hatched, and the second analyzed the number of chicks that successfully fledged. In both models, fixed effects included treatment (ant exclusion vs. control), laying date (continuous), their interaction, and the time interval between egg laying of the first egg and treatment assignment in each nest (laying date-treatment interval hereafter).

For mite infestation intensity, we fitted a mixed-effects ordinal regression model using the PROC GLIMMIX procedure with a cumulative logit link function and multinomial error distribution. The response variable was mite intensity, treated as an ordinal variable reflecting increasing levels of ectoparasite load (see Methods for details). For analyses of chick body mass and wing length, we used linear mixed-effects models with Gaussian error distributions and identity link functions, also implemented with PROC GLIMMIX. In all mixed models, fixed effects included treatment, laying date, laying date-treatment interval, and chick tarsus length to control for structural body size as covariates. To account for non-independence among chicks from the same nest, nest ID was included as a random intercept. When a significant interaction between laying date and treatment was found, separate models were run for control and ant-exclusion nests using the same covariance structure to clarify group-specific patterns. Excluding the three nests from the ant-exclusion treatment where ants were present (see Results and Discussion) did not change the results; treatment and its interaction with laying date significantly affected mite infestation, weight, and wing length, so all nests were retained in the analyses.

We used a general linear model (PROC GLM) to analyze variation in the heterophil-to-lymphocyte (H/L) ratio, with treatment, laying date, and nestling body weight as fixed effects. The interaction between treatment and laying date was initially included but removed due to its lack of significance and the limited sample size, to reduce overfitting and improve model parsimony^60^. A second GLM was conducted to test the predicted linear relationship between the H/L ratio and mite infestation intensity, treating mite intensity as a continuous predictor. This specific analysis allowed us to evaluate the a priori expectation that higher mite infestation levels are associated with increased physiological stress in house sparrow nestlings. Standard model validation graphs^61^ confirmed that the assumptions of homogeneity of variance and normality of residuals were fulfilled.

Finally, to assess the direct and indirect effects of ant exclusion on nestling condition and mite infestation, we implemented a piecewise SEM using the piecewiseSEM package in R (v4.3.2)^62^. This approach allowed us to explicitly test causal relationships and disentangle direct *versus* indirect effects, which cannot be fully established when fitting models separately. Specifically, we evaluated whether ant exclusion influenced chick body mass directly and/or indirectly through mite intensity. The SEM consisted of two linear mixed models (Fig. 4). The first model included chick body mass as the response variable, with ant exclusion (as yes *versus* no), laying date, and mite intensity as predictors. Nest ID was incorporated as a random intercept to account for potential non-independence among chicks from the same nest. The second model treated mite intensity as the response, with ant exclusion and laying date as explanatory variables, also including nest ID as a random intercept.

## Supplementary information

Supplementary Information

Description of Additional Supplementary Files

Supplementary Data 1

Related Manuscript File- nr-reporting-summary filled

## Data Availability

Raw data for analyses and figures are available in Zenodo at 10.5281/zenodo.15721917. All other data are available from the corresponding author on reasonable request.

## Code availability

Codes for analyses and figures are available in Zenodo at 10.5281/zenodo.15721917.
