# Supplementary material for: Phenology modulates the top-down control of ants on bird ectoparasites: from mutualism to antagonism
Source: Commun Biol. 2025 Dec 22;8:1831. doi: 10.1038/s42003-025-09387-9 (PMC12749245; doi:10.1038/s42003-025-09387-9)
Supplement: Supplementary file 1 — Supplementary Information [file 42003_2025_9387_MOESM1_ESM.pdf]

Appendix S1

Phenology Modulates the Top-Down Control of Ants on Bird Ectoparasites: From Mutualism to Antagonism

Avilés, J.M., Salido, A., Reyes-López, J. L. and Parejo, D.

Supplementary Table 1. Effect of ant exclusion on house sparrow hatching and fledging success. Results of the Poisson GLMs assessing the number of hatchlings and the number of fledglings in relation to treatment, laying date and laying date-treatment interval. The models also include the interaction between treatment and laying date. Analyses were based on the 54 nests in which at least one egg hatched and the 53 nests from which at least one chick fledged, respectively.

**Response variable**	**Fixed Effect**	**Estimate**	**SE**	**DF**	**t / F**	**p**	**95% CI (Lower – Upper)**
**Number of hatchlings**	Intercept	1.412	0.635	49	2.22	0.031	0.136 – 2.689
	Treatment† (control)	–0.302	0.908	49	0.11	0.741	–2.126 – 1.522
	Laying date	0.0007	0.006	49	0.20	0.913	–0.012 – 0.013
	Laying date × Treatment	0.003	0.009	49	0.10	0.751	–0.015 – 0.019
	Laying date-Treatment interval	0.004	0.010	49	0.23	0.63	-0.015– 0.025
**Number of fledglings**	Intercept	1.294	0.643	48	2.01	0.050	0.002 – 2.586
	Treatment† (control)	0.051	0.920	48	0.00	0.956	–1.798 – 1.901
	Laying date	0.002	0.006	48	0.09	0.760	–0.011 – 0.014
	Laying date × Treatment	–0.001	0.009	48	0.01	0.904	–0.019 – 0.016
	Laying date-Treatment interval	0.004	0.010	48	0.15	0.699	-0.017– 0.025

†The reference category for the treatment variable was “ant exclusion.”

Supplementary Table 2. Effect of ant presence on house sparrow mite infestation. Results of the ordinal mixed-effects regression model assessing mite infestation intensity in relation to treatment, laying date, laying date-treatment interval and tarsus length. The model also includes the interaction between treatment and laying date and the nest as a random intercept to account for non-independence among chicks within the same nest. Significant terms are highlighted in bold. Mite intensity was scored using a four-point ordinal scale: 1 = no mites, 2 = 1–10 mites, 3 = 20–30 mites, and 4 = >30 mites.

**Fixed Effect**	**Estimate**	**SE**	**DF**	***t / F***	***p***	**95% CL (Lower – Upper)**
Intercept (Category 1)	-1.09	7.79	188	–0.14	0.889	–16.46 – 14.27
Intercept (Category 2)	2.45	7.82	188	0.31	0.755	–12.96 – 17.86
Intercept (Category 3)	5.03	7.84	188	0.64	0.522	–10.44 – 20.49
**Treatment† (control)**	25.14	9.12	46.58	**7.60**	**0.008**	**6.79 – 43.49**
**Laying date**	0.01	0.03	60.29	**4.34**	**0.043**	**–0.05 – 0.07**
**Interaction Treatment x Laying date**	-0.21	0.08	43.65	**6.36**	**0.015**	**–0.38 – –0.04**
Laying date-Treatment interval	-0.05	0.08	17.9	0.46	0.508	–0.22 – 0.11
Tarsus length	-0.09	0.34	188	0.09	0.770	–0.76 – 0.56

**Ramdom effect**	**Estimate**	**SE**	***Z***	***p***	**95% CL (Lower – Upper)**
**Nest ID**	9.05	3.45	**2.62**	**0.004**	**4.82 – 22.72**

†The reference category for the treatment variable was “ant exclusion.”

Supplementary Table 3. Effect of ant exclusion on house sparrow chick body mass. Results of the linear mixed-effects model assessing chick weight in relation to treatment, laying date, treatment-laying date interval, and tarsus length. The model also includes the interaction between treatment and laying date and the nest as a random intercept to account for non-independence among chicks within the same nest. Significant terms are highlighted in bold.

**Fixed Effect**	**Estimate**	**SE**	**DF**	**t / F**	***p***	**95% CL (Lower – Upper)**
Intercept	25.2	3.15	187.2	8.00	<0.0001	18.99 – 31.41
**Treatment† (control)**	10.44	2.73	158.8	**14.67**	**0.0002**	**5.06 – 15.83**
**Laying date**	-0.028	0.02	107.7	**28.43**	**<0.0001**	**-0.068 – 0.012**
**Interaction Treatment x Laying date**	-0.099	0.026	169.1	**14.58**	**0.0002**	**-0.150 – -0.048**
Laying date-Treatment interval	-0.051	0.048	35.52	1.11	0.3	-0.148 – 0.047
Tarsus length	0.092	0.115	162.5	0.64	0.426	-0.135 – 0.319
**Ramdom effect**	**Estimate**	**SE**	**Z**	***p* valor**		**95% CI (Lower – Upper)**
**Nest ID**	3.68	0.96	**3.82**	**<0.0001**		**2.33 – 6.62**

†The reference category for the treatment variable was “ant exclusion.”

Supplementary Table 4. Effect of ant exclusion on house sparrow chick wing length. Results of the linear mixed-effects model assessing wing length in relation to treatment, laying date, their interaction, treatment–laying date interval, and tarsus length. The model also includes the interaction between treatment and laying date and the nest as a random intercept to account for non-independence among chicks within the same nest. Significant terms are highlighted in bold.

**Fixed Effect**	**Estimate**	**SE**	**DF**	**t / F**	***p***	**95% CL (Lower – Upper)**
Intercept	2.72	0.86	176.9	3.15	0.0019	1.02 – 4.43
**Treatment (control)**	3.07	0.76	159.1	**16.18**	**<.0001**	**1.56 – 4.58**
Laying date	0.017	0.0059	106.7	0.75	0.38	0.006 – 0.029
**Interaction Treatment x Laying date**	–0.028	0.0071	172	**15.44**	**0.0001**	**–0.042 – –0.014**
Laying date-Treatment interval	–0.0049	0.0158	22.8	0.09	0.761	–0.038 – 0.028
Tarsus length	0.0378	0.0297	133	1.62	0.2052	–0.021 – 0.097

**Ramdom effect**	**Estimate**	**SE**	**Z**	***p* valor**	**95% CI (Lower – Upper)**
**Nest ID**	0.40	0.126	**3.17**	**0.001**	**0.234 – 0.831**

†The reference category for the treatment variable was “ant exclusion.”

Supplementary Table 5. Effect of Ant Exclusion on House Sparrow Chick H/L Ratio. Results of the General linear model that assesses the H/L ratio in relation to treatment, laying date, and chick weight.

Fixed Effect	Estimate	SE	DF	*t / F*	*p*	95% CI (Lower – Upper)
Intercept	-0.090	0.427	34	-0.21	0.834	-0.957 – 0.777
**Treatment† (control)**	**-0.123**	**0.056**	**34**	**2.18**	**0.036**	**-0.238 – -0.008**
**Laying date**	**0.0074**	**0.0022**	**34**	**3.36**	**0.0019**	**0.0029 – 0.0119**
Weight	-0.018	0.011	34	-1.62	0.114	-0.041 – 0.0046

†The reference category for the treatment variable was “ant exclusion.”
