# Supplementary material for: Phenology modulates the top-down control of ants on bird ectoparasites: from mutualism to antagonism
Source: Commun Biol. 2025 Dec 22;8:1831. doi: 10.1038/s42003-025-09387-9 (PMC12749245; doi:10.1038/s42003-025-09387-9)
Supplement: Supplementary file 2 — Description of Additional Supplementary Files [file 42003_2025_9387_MOESM2_ESM.pdf]

Description of Additional Supplementary Files:

File: Supplementary Data 1

Description: An Excel file is included with two columns: the first one (next-box) identifies the name assigned to the nest box, and the second one (clutch_size) contains the number of sparrow eggs in each box.
